# Sandwich Immunosensor Based on Particle Motion: How Do Reactant Concentrations and Reaction Pathways Determine the Time-Dependent Response of the Sensor?

**DOI:** 10.1021/acssensors.3c01549

**Published:** 2023-11-13

**Authors:** Claire M. S. Michielsen, Alissa D. Buskermolen, Arthur M. de Jong, Menno W. J. Prins

**Affiliations:** † Department of Biomedical Engineering, Eindhoven University of Technology, Eindhoven 5612 AE, The Netherlands; ‡ Department of Applied Physics, Eindhoven University of Technology, Eindhoven 5612 AE, The Netherlands; § Institute for Complex Molecular Systems (ICMS), Eindhoven University of Technology, Eindhoven 5612 AE, The Netherlands; ∥ Helia Biomonitoring, Eindhoven 5612 AR, The Netherlands

**Keywords:** sandwich immunosensor, particle-based biosensing, biosensor kinetics, response time, binder densities, reaction pathways

## Abstract

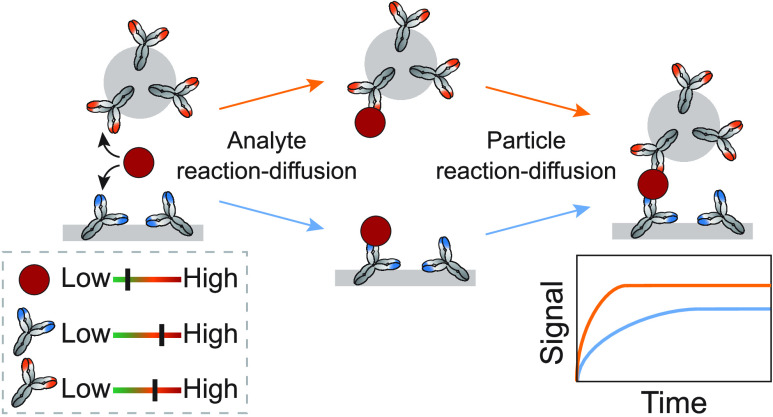

To control and optimize the speed of a molecular biosensor, it is crucial to quantify and understand the mechanisms that underlie the time-dependent response of the sensor. Here, we study how the kinetic properties of a particle-based sandwich immunosensor depend on underlying parameters, such as reactant concentrations and the size of the reaction chamber. The data of the measured sensor responses could be fitted with single-exponential curves, with characteristic response times that depend on the analyte concentration and the binder concentrations on the particle and substrate. By comparing characteristic response times at different incubation configurations, the data clarifies how two distinct reaction pathways play a role in the sandwich immunosensor, namely, analyte binding first to particles and thereafter to the substrate, and analyte binding first to the substrate and thereafter to a particle. For a concrete biosensor design, we found that the biosensor is dominated by the reaction pathway where analyte molecules bind first to the substrate and thereafter to a particle. Within this pathway, the binding of a particle to the substrate-bound analyte dominates the sensor response time. Thus, the probability of a particle interacting with the substrate was identified as the main direction to improve the speed of the biosensor while maintaining good sensitivity. We expect that the developed immunosensor and research methodology can be generally applied to understand the reaction mechanisms and optimize the kinetic properties of sandwich immunosensors with particle labels.

Particles are widely used as detection labels in affinity-based biosensors because particles are easily biofunctionalized, are very stable, and give large signals for easy detection, using optical methods for example.^1−3^ Particle labels are applied in lateral flow strips,^4^ plasmonic biosensors,^5^ microfluidic sensors with magnetic actuation,^6−8^ biosensors based on particle aggregation,^9^ and flow cell biosensors with single-molecule resolution.^10−13^ In these biosensors, a wide variety of particle types are used with sizes ranging from nanometers to micrometers. An important aspect of the design of a biosensor is to optimize its response time because that determines if a biosensor can be used in time-critical applications that can tolerate only a short delay between measurement and resulting follow-up actions, such as point-of-care testing of acute patient conditions and the monitoring and control of fluctuations in bioprocesses.

Previous papers have studied in detail the kinetics of two-component reactions, where analyte molecules bind to affinity molecules that are immobilized on a sensing surface, highlighting the roles of reactant concentrations, diffusion, and flow properties of the sensor.^14,15^ However, analytes at low concentrations are generally measured in a sandwich configuration, which is a more intricate three-component arrangement in which analyte molecules are captured between two affinity molecules. Only a few studies have been reported on the kinetics of sandwich sensors.^16−18^ These studies focused on a step-by-step approach where the analyte is initially exposed to a first antibody and later to a second antibody. However, for an optimal speed of a sensor with minimal fluid manipulations, it is advantageous to expose the analyte molecules to both antibodies simultaneously in order to achieve a fast formation of sandwich complexes in the biosensor.

In this article, we experimentally study the mechanisms that underlie the kinetics of a sandwich immunosensor with particle labels, where both binders interact *simultaneously* with the analyte molecules. The studied sensor is based on biofunctionalized particles that move freely over a biofunctionalized substrate, called Biosensing by Particle Motion (BPM).^10^ Antibodies are used for the biofunctionalizations—hence immunosensor—because antibodies have strong and specific binding properties and are available for a wide range of biomarkers.^19^ Lactoferrin is used as the model analyte. The response of the sensor is studied as a function of time and three characteristic properties of the binding curves are extracted: the maximal signal, the characteristic time to reach the maximal signal, and the initial slope of the signal. These parameters are studied as a function of the analyte concentration, antibody density, and height of the sensor chamber. Experiments with preincubation of the analyte with either the substrate or the particles were used to zoom in on the different reaction pathways of the sandwich complex formation. This provides a generalizable methodology to distinguish reaction pathways, identify which pathway dominates the response time of the biosensor, and enhance the response time of sandwich immunosensors with simultaneous antibody exposure.

## Results and Discussion

### Sandwich Immunosensing Using Biosensing by Particle Motion

**Figure 1:**
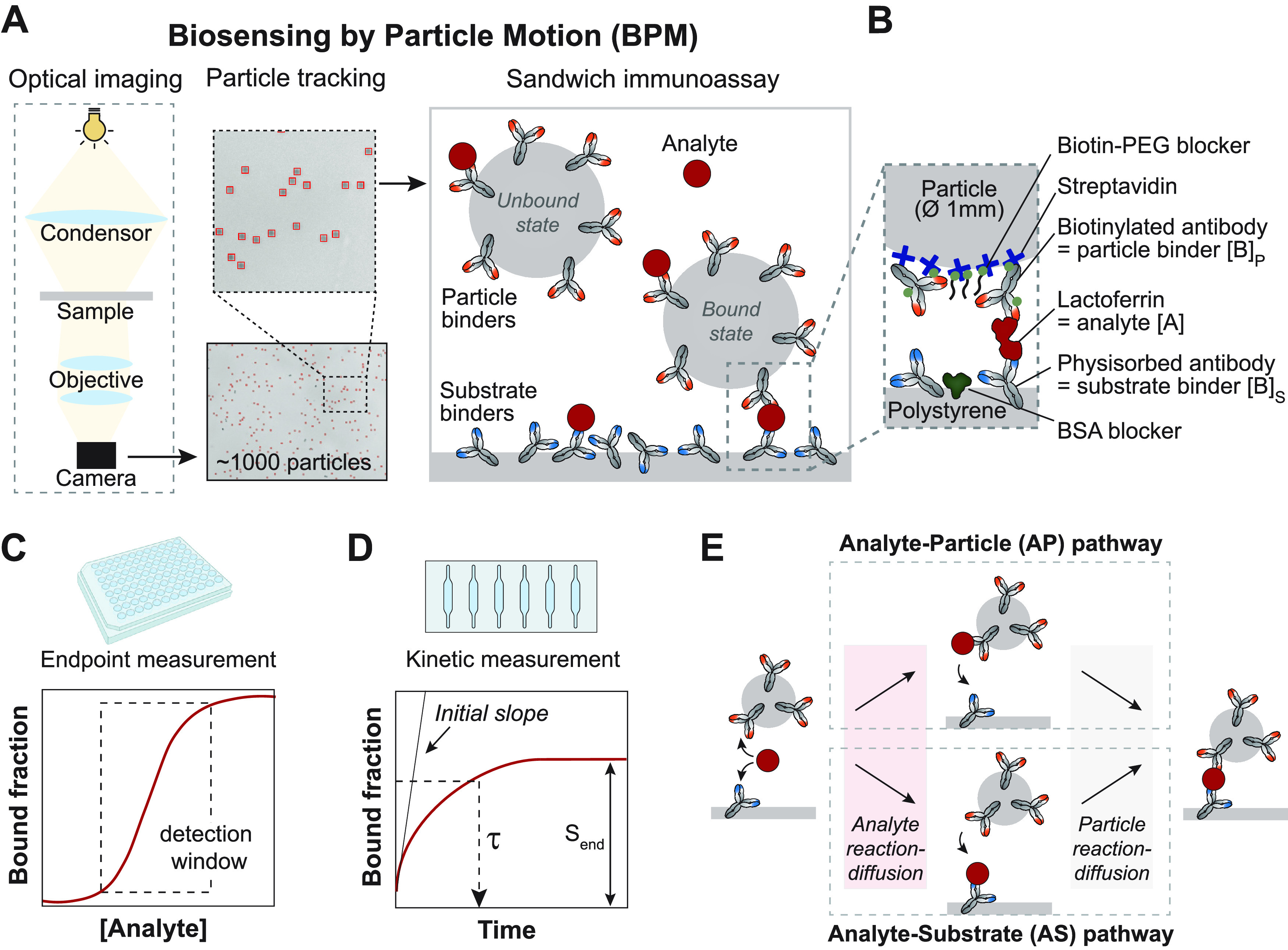
Sandwich immunosensing using Biosensing by Particle Motion. (A) Schematic representation of Biosensing by Particle Motion. Particles are tracked over time using video microscopy with particle identification and tracking software.^20^ In the presence of the analyte, the antibodies on the substrate and on the particles bind to the analyte and form a sandwich bond, resulting in a bound state of the particles. The sketch is not to scale; the particles are much larger than the molecules. (B) Close-up of the molecular components of the BPM immunosensor, with bovine lactoferrin as the analyte molecule. Antibodies are physisorbed on a polystyrene substrate, and open areas are blocked with bovine serum albumin (BSA). Streptavidin-coated particles (1 μm diameter) are functionalized with biotinylated antibodies and blocked with biotin-PEG. (C) The bound fraction was measured for varying analyte concentrations using endpoint measurements in 96-well plates. (D) The signal response over time was measured by using flow cells. Upon addition of the analyte, the bound fraction increases over time until a plateau is reached. In this study, the factors are investigated that contribute to the characteristic response time (τ), maximal signal (*S*_end_), and initial slope. (E) The molecular sandwich complex between particle and substrate can be formed via two pathways. Pathway AP: an analyte molecule is first captured by an antibody on a particle and subsequently by an antibody on the substrate. Pathway AS: an analyte molecule is first captured by an antibody on the substrate and, subsequently, by an antibody on a particle. Analyte reaction-diffusion plays a role in the first processes (A → AP and A → AS) and particle reaction-diffusion plays a role in the second processes (AP → SAP and AS → SAP).

The sandwich immunosensor used in this study is sketched in Figure 1A. The sensor contains an antibody-functionalized substrate and hundreds to thousands of antibody-functionalized particles with a diameter of 1 μm. The particles remain in close proximity to the substrate due to gravitational forces; the average distance between particle and substrate is about 1 μm.^10^ In the absence of analyte, the particles diffuse freely over the substrate, which is referred to as the unbound state. When the analyte is present in solution, the analyte can bind to antibodies on the particles and antibodies on the substrate, causing sandwich bonds between the particle and substrate. A single sandwich bond between a particle and the substrate restricts the motion of the particle, which is referred to as the bound state. The motion behavior of hundreds to thousands of particles is recorded as a function of time by using video microscopy. The time-dependent readout parameter used in this study is the bound fraction, i.e., the ratio between the population of bound states and the total number of states during the measurement time (see Supporting Information Figure S1).

Figure 1B sketches the molecular architecture of the studied sandwich sensor, with bovine lactoferrin as the analyte and polyclonal anti-lactoferrin antibodies as the binders on the particles and the substrate. Lactoferrin is an 80 kDa iron-binding glycoprotein that supports the immune system and is present in secretory fluids. Two experimental setups were used to develop and study the lactoferrin sensor. First, endpoint measurements were performed in 96-well plates to screen conditions, such as the antibody densities on particles and substrate (Figure 1C). Thereafter, kinetic measurements were performed in flow cells to study the sensor response as a function of time (Figure 1D). The data were fitted with single-exponential curves with a signal change Δ*S* and a characteristic response time τ
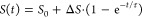
1with *S*_0_ the bound fraction signal at *t* = 0. This equation describes the response of the biosensor system to a perturbation: the system starts with initial signal *S*_0_, is perturbed at *t* = 0 by a step function in the analyte concentration, and then develops single-exponentially and asymptotically with a characteristic time τ toward a final state with signal *S*_end_ = *S*_0_ + Δ*S*. The kinetics of the sensor were studied by fitting the initial slopes of the measured signal-versus-time curves, and by fitting the full curves using eq 1.

In the sensor, the particles transition from unbound to bound states by molecular sandwich formation, which can occur via two pathways, as sketched in Figure 1E. A first pathway for sandwich formation is that an analyte molecule diffuses through the solution, is captured by an antibody on a particle, and is thereafter captured by an antibody on the substrate, referred to as the AP pathway (analyte is first captured by a particle). A second pathway is that an analyte molecule diffuses through the solution, is captured by an antibody on the substrate, and is thereafter captured by an antibody on a particle, referred to as the AS pathway (analyte is first captured by the substrate). The reaction-diffusion of analyte plays a role in the first parts of both pathways, while the reaction-diffusion of the particles plays a role in the second parts.

It is not a priori clear if and when pathway AP or AS dominates the signal and the response time of the sensor. This could depend, for example, on the sensor geometry (volume of the measurement chamber, number of particles) and the antibody density on the particles and substrate. In the studied sensor, the total number of antibodies on the substrate is orders of magnitude higher than the total number of antibodies on the particles, due to the large difference of total surface area, caused by the low coverage of the substrate by particles (see Supporting Information Table S1). Therefore, at equal areal binder densities, a random free analyte molecule in solution has a higher probability of being captured by an antibody on the substrate (A → AS) than by an antibody on a particle (A → AP). However, an analyte–particle complex is in very close proximity to the substrate, causing a high sandwich reaction rate (AP → SAP), while on average, it takes much more time for a particle to encounter an analyte molecule on the substrate, causing a lower sandwich reaction rate (AS → SAP). To unravel the mechanisms that underlie the speed of the sensor response, the time dependence of the sensor signal was studied as a function of analyte concentration, binder density, measurement chamber height, and different incubation protocols, as described in the next sections.

### Optimization of Antibody Density on Particles

**Figure 2:**
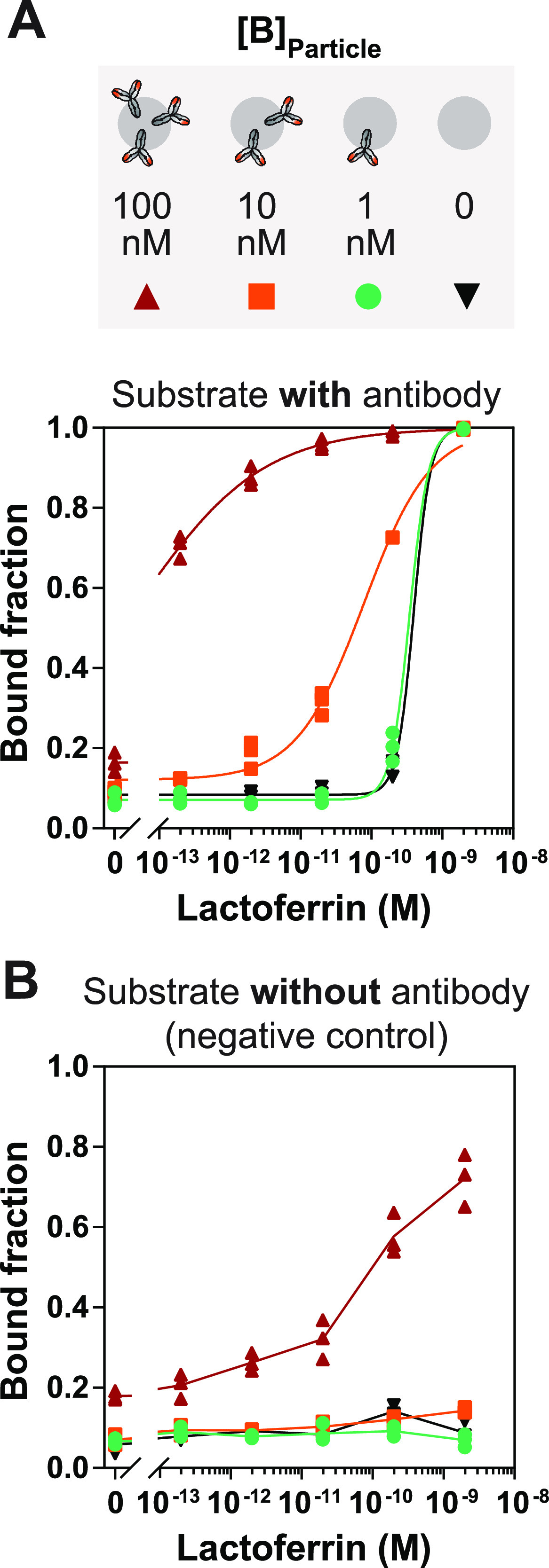
Dose–response curves for different antibody densities on the particles, measured in 96-well plates. The streptavidin-coated particles were functionalized with biotinylated antibodies at the given antibody concentrations: [B]_Particle_ = 100 nM (red), 10 nM (orange), 1 nM (green), and no antibodies (black). (A) Dose–response curves of the differently functionalized particles on an antibody-functionalized substrate (50 nM). (B) Dose–response curves of the particles on a substrate without antibodies (only BSA; negative control). All measurements were performed in triplicate (symbols of all three measurements are shown), and the solid lines are a guide to the eye.

In the lactoferrin BPM sandwich immunosensor, anti-lactoferrin antibodies are immobilized on the substrate and on the particles. In the presence of lactoferrin, the particles can form a sandwich complex with the binders on the substrate, resulting in an increased bound fraction for increasing lactoferrin concentrations (Figure 1C). It is important to optimize the antibody immobilization processes and minimize nonspecific interactions between particles and substrate. To study these interactions, particles were prepared with different concentrations of antibodies ([B]_Particle_) and the dependence of the signal on the lactoferrin concentration was studied with and without antibodies on the substrate (Figure 2A,B). Four different particle binder concentrations were tested, including a negative control without binder molecules. The highest antibody density on the particles resulted in the lowest detection range (below picomolar). For lower antibody densities, the detection range shifts toward picomolar concentrations. For the highest lactoferrin concentrations, negative controls with antibodies on only one side (i.e., antibodies on the substrate, but none on the particles, in Figure 2A; or high density of antibody on the particles, but none on the substrate, in Figure 2B) give a high bound fraction. These one-sided negative controls indicate that high lactoferrin concentrations can cause significant nonspecific interactions with antibody-free particles as well as an antibody-free substrate. To limit signals due to nonspecific interactions, the standard condition selected for this research was to functionalize the particles with 10 nM antibody (cf. the orange data points) and use lactoferrin concentrations in solution always lower than 250 pM.

### Influence of Analyte and Substrate Binder Concentrations on Sensor Response

**Figure 3:**
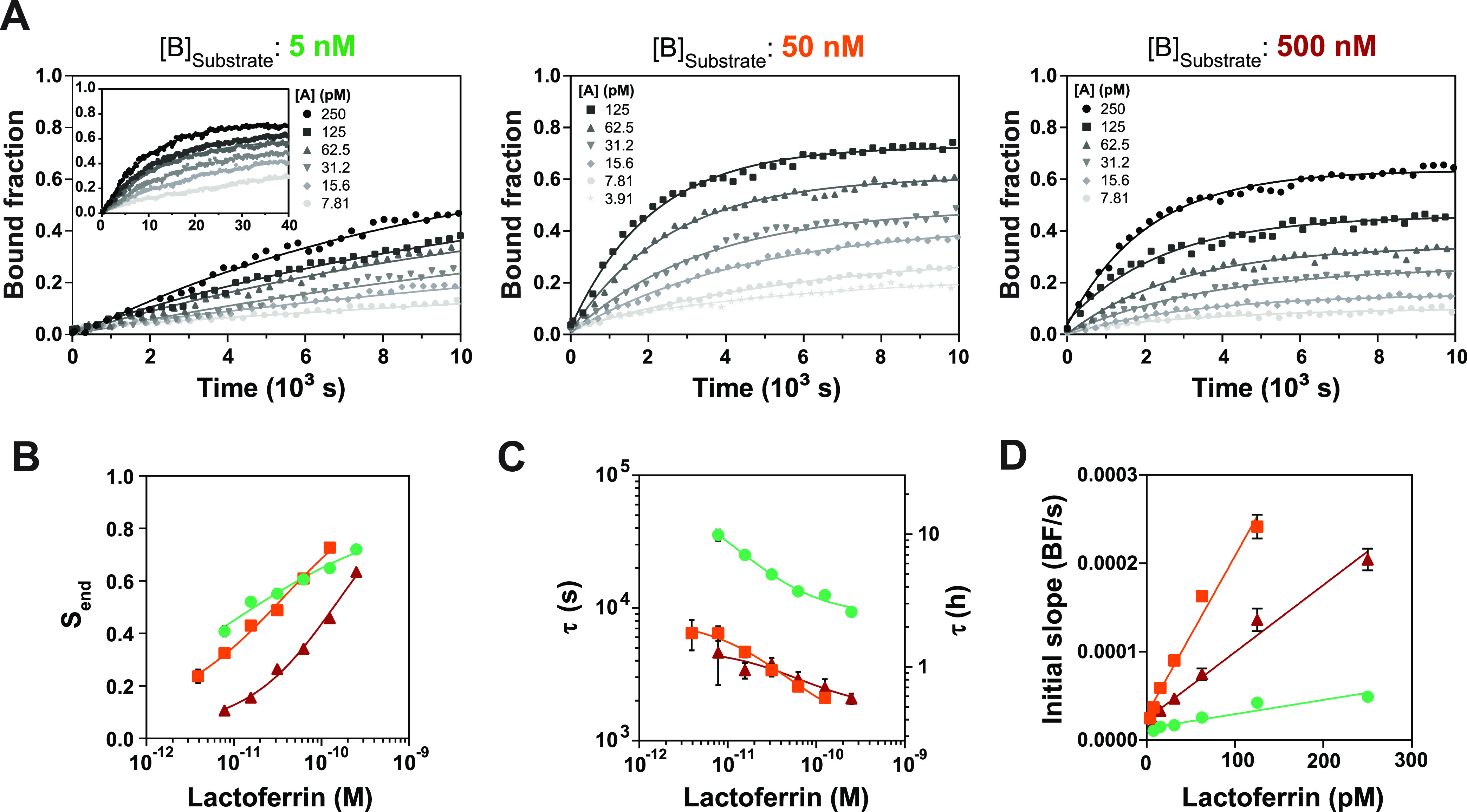
Influence of the analyte concentration and the substrate binder concentration on the sensor response. (A) The signal of the BPM sensor as a function of time was studied in a static flow cell for different lactoferrin concentrations. The polystyrene substrates were functionalized by physisorption of antibodies using concentrations of 5 nM (green), 50 nM (orange), and 500 nM (red). The data were fitted with single-exponential curves according to eq 1. (B) The maximal signal *S*_end_ was extracted from the fits in (A). (C) The characteristic response time τ extracted from the fits in (A) plotted on log–log scales. The τ values show a slope of roughly minus 1/3, which means that τ scales roughly as τ ∝ [A]^−1/3^. (D) The initial slope (bound fraction per second) extracted from linear fits of the first 2000 s (Supporting Information Figure S4). The solid lines in (B–D) are a guide to the eye and the error bars (not always visible) indicate the 95% confidence interval of the values extracted from the fits.

The kinetics of the immunosensor were studied by monitoring the sensor signal as a function of time in a flow cell, using the conditions determined in Figure 2. Different analyte concentrations were added to separate flow cells containing the particles on a biofunctionalized substrate. The particles were functionalized with 10 nM biotinylated antibodies, which gives a maximum binder density of approximately 600 binders per particle and a maximum total amount of 4 × 10^7^ particle-coupled binders in the flow cell (Supporting Information Table S1). The analyte is added to the particles and substrate at the same time, which is referred to as simultaneous incubation. After the addition of the analyte, measurements of the bound fraction were performed as a function of time, in the absence of any flow; see Figure 3. Analyte concentrations were varied for three different substrate binder densities, obtained by physisorption of 5 nM (green), 50 nM (orange), and 500 nM (red) antibody. The addition of 5, 50, and 500 nM binders to the flow cell translates to a maximum of 6 × 10^10^, 6 × 10^11^, and 6 × 10^12^ antibody molecules in the flow cell, respectively. The negative control is shown in Supporting Information Figure S2. The data show that the sensor response systematically depends on the reactant concentrations that were varied, namely, the analyte concentration and the physisorbed antibody concentration.

Figure 3A shows the measured data as symbols, and the fits according to eq 1 as continuous lines. The graph shows that all measured time-dependent curves behave according to the single-exponential response given in eq 1. We attribute the sensor response to the molecular binding pathways as sketched in Figure 1E. The sketch shows unidirectional processes only, without any reversibility, because the used polyclonal anti-lactoferrin antibodies appear to bind very strongly to the analyte. Reversible binding, particle dissociation, and decreases of bound fraction have not been observed in the experiments (see Supporting Information Figure S3). Thus, the effective reaction rate constants that underlie the observed time profiles are caused by molecular association processes only. Four association processes take place in the sensor, as shown in Figure 1E: binding of free analyte to antibodies on the substrate, binding of free analyte to antibodies on particles, binding of particle-bound analyte to antibodies on the substrate, and binding of substrate-bound analyte to antibodies on the particles. The bound fraction signal is generated by two serial binding processes that occur in parallel (pathways AP and AS). Interestingly, this complex serial-parallel reaction configuration causes a single-exponential behavior for all conditions studied, since all measured curves follow the behavior of eq 1. This brings the question: which of these processes is dominant for the response of the immunosensor?

All experiments show that when the analyte concentration increases, the maximal signal increases (Figure 3B), the characteristic response time τ decreases (Figure 3C), and the initial slope increases (Figure 3D). Thus, higher analyte concentrations result in higher signals and a faster sensor response for all sensor substrates that were prepared.

A comparison of sensors with different substrate preparations gives insights into the mechanisms that play a role in the kinetics of the immunosensor. First of all, increasing the binder concentration from 5 to 50 nM results in similar maximal signal values (Figure 3B), a decrease of τ by roughly a factor 6 (Figure 3C) and an increase of initial slope by about a factor 5 (Figure 3D). This indicates that the sensor with more antibodies on the substrate responds faster, while the final signal value is the same. The fact that for different amounts of antibodies equal maximal signal values are observed, suggests that the sensor operates in a binder-dominated regime with analyte depletion.^15^ In that regime, the sensor chamber contains fewer analyte molecules than antibodies, causing all analyte molecules to be captured from solution. This results in a maximal signal that is independent of the total number of antibodies in the sensor. Still, the characteristic response time would decrease and the initial slope would increase with the number of active binders in the sensor, which is indeed observed in the experiments of Figure 3. Equal maximal signals can also occur when the sensor is dominated by the AP pathway. In that case, the response time would decrease with higher substrate binder density due to the higher probability that an analyte-bound particle can form a sandwich complex. We will discuss the AS versus AP pathway comparison in a later section of this paper.

Increasing the binder concentration from 50 to 500 nM results in decreased maximal signal values by a factor of 2 (Figure 3B), similar τ values (Figure 3C) and decreased initial slopes (Figure 3D). Apparently, an even higher density of antibodies on the substrate results in a less sensitive and slower immunosensor. Several hypotheses can be considered to explain these observations. (1) A high binder density may cause steric hindrance, resulting in reduced accessibility of binder molecules for the formation of a sandwich complex. However, we will show in preincubation experiments that the substrate binders are still accessible for analyte-bound particles, which invalidates the steric hindrance hypothesis. (2) For higher antibody densities on the substrate and the same antibody density on the particle, more analyte molecules can bind to the substrate first, which would lead to pathway AS becoming dominant over pathway AP. Analyte molecules bound to the substrate are less effectively detected, since the particles probe only a limited area of the substrate during the measurement time, resulting in a lower sensitivity and slower response time. However, experiments in the subsequent sections will show that the AS pathway is already dominant for substrates prepared with 50 nM binders, which invalidates the pathway shift hypothesis. (3) A high binder density results in faster analyte depletion by the substrate. Since the sensing surface area of the flow cell is much larger than the field of view of the detection area, analyte molecules would be captured from solution before they reach the detection area, resulting in a lower and slower sensor response. This hypothesis is further studied and supported in Supporting Information Figure S7.

The observations obtained by varying the substrate binder density of the sensor indicate that different processes contribute to the sensor response. From these results, we conclude that the 50 nM substrate condition is optimal, as it gives a sensitive sensor response (Figure 3B), a fast characteristic response time (Figure 3C), and a high value for the initial slope (Figure 3D). In the time-dependent response of the immunosensor, two diffusion processes also may play a role: diffusion of analyte in the solution and diffusion of particles over the substrate (see Figure 1E); these processes will be examined in the subsequent sections.

### Role of Analyte Diffusion in the Sensor Response

**Figure 4:**
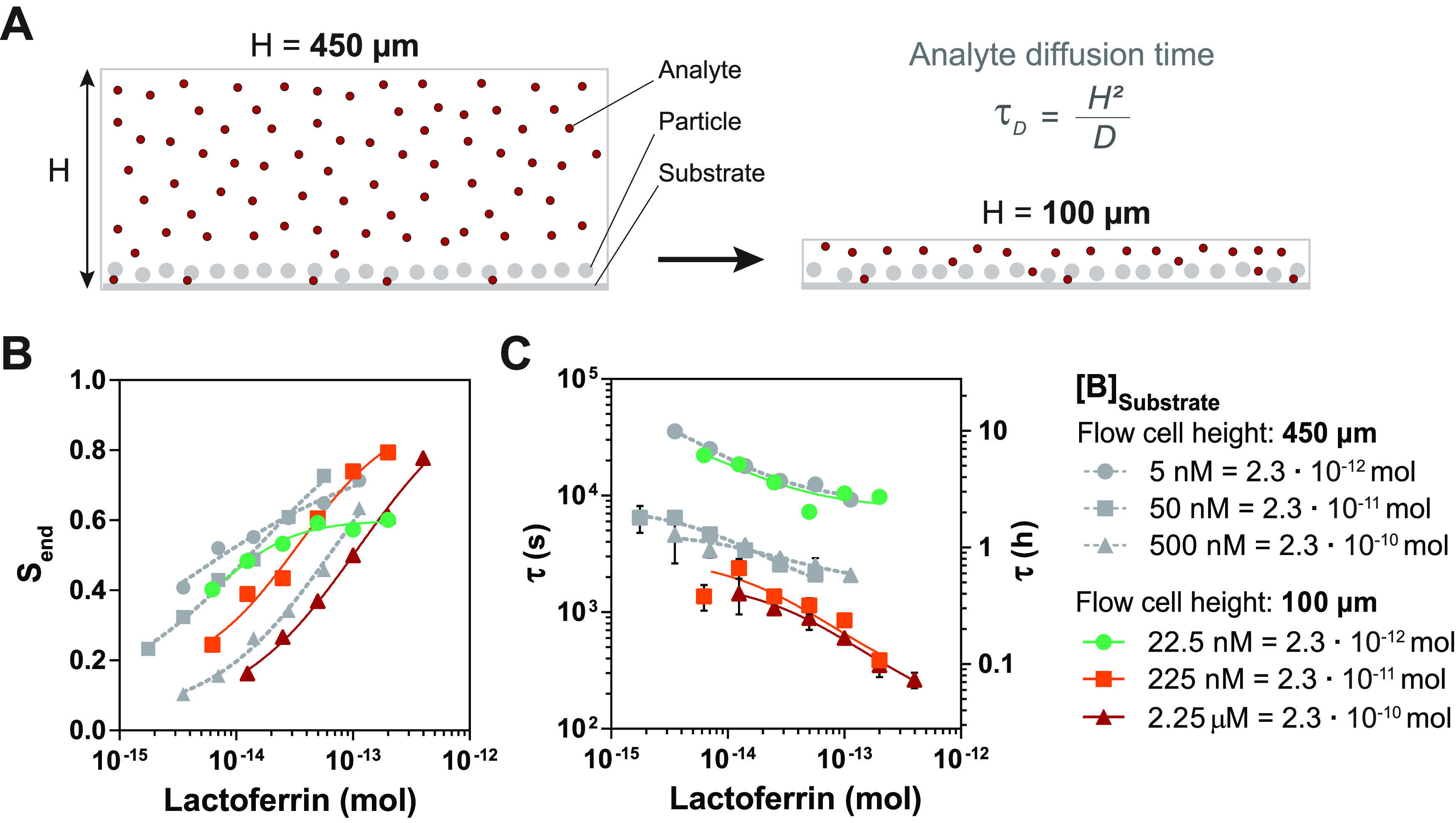
Role of analyte diffusion in the sensor response, studied with different flow cell heights. (A) A reduction of the flow cell height H from 450 to 100 μm reduces the distance that analyte molecules need to diffuse to reach the sensing surface. This theoretically leads to a (4.5)^2^ = 20 times faster analyte diffusion time (τ_D_). (B) The maximal signal *S*_end_ and (C) the characteristic response time τ as a function of the number of lactoferrin molecules in the sensor chamber, for *H* = 450 μm (gray) and *H* = 100 μm (colors). The measured signal as a function of time with single-exponential fits is shown in Supporting Figure S6. Lines are a guide to the eye, and the error bars (not always visible) indicate the 95% confidence interval of the values extracted from the single-exponential fit of the time profiles.

To study the role of diffusion of analyte molecules in the sensor response, sensor chambers with different heights were studied. The data in Figure 3 were recorded with a flow cell height of 450 μm. In Figure 4, results from flow cells with a chamber height of 100 μm are compared to those from flow cells with a height of 450 μm. A lower height reduces the average distance that analyte molecules need to diffuse to reach binders on the substrate or on the sedimented particles. Therefore, a low flow cell height would result in a faster characteristic response time in case analyte diffusion plays a significant role in the sensor response. As the molecular diffusion time scales with the square of the distance, the characteristic response time of a sensor with a flow cell height of 100 μm could potentially be (4.5)^2^ = 20 times faster than a sensor with a flow cell height of 450 μm.^14,15^ The 100 μm flow cells were prepared with antibody concentrations higher than those of the 450 μm flow cells. The functionalization concentration was chosen to have equal total numbers of antibodies in the measurement chamber for the two chamber heights in order to aim for similar immobilized antibody surface densities. Subsequently, the signal responses over time were measured for varying analyte concentrations (full signal response over time curves and single-exponential fits are shown in Supporting Information Figure S6). Since analyte depletion occurs, the sensor response is expected to depend on the number of analyte molecules in the measurement chamber rather than on the concentration of analyte molecules. Therefore, the maximal signal and characteristic response time are plotted as a function of the number of moles instead of molar concentration. Figure 4B shows that the dose–response curves for the three different substrates in the 100 μm flow cell are similar, with a slight shift to the right, indicating that the substrate binder densities may indeed be quite similar. Furthermore, the slopes and trends as a function of substrate biofunctionalization concentration are similar, confirming that the data obtained with the 100 μm flow cells can be compared to the data obtained with the 450 μm flow cells.

Interestingly, the trend in the characteristic response time of the lowest substrate binder density (green data) is the same for both flow cells (Figure 4C). This indicates that for the low substrate binder density, analyte diffusion does not affect the signal response over time of the sensor. The higher substrate binder densities (orange squares vs gray squares, red triangles vs gray triangles) both show a faster response by a factor 2 to 3 for the 100 μm flow cells, but certainly not a factor 20 (Figure 4C). This proves that analyte diffusion contributes somewhat to the sensor response but only for the conditions of the higher substrate binder densities. In the next section, we will investigate which pathway is dominant in the immunosensor.

### Distinguishing the Reaction Pathways of the Sandwich Immunosensor

**Figure 5:**
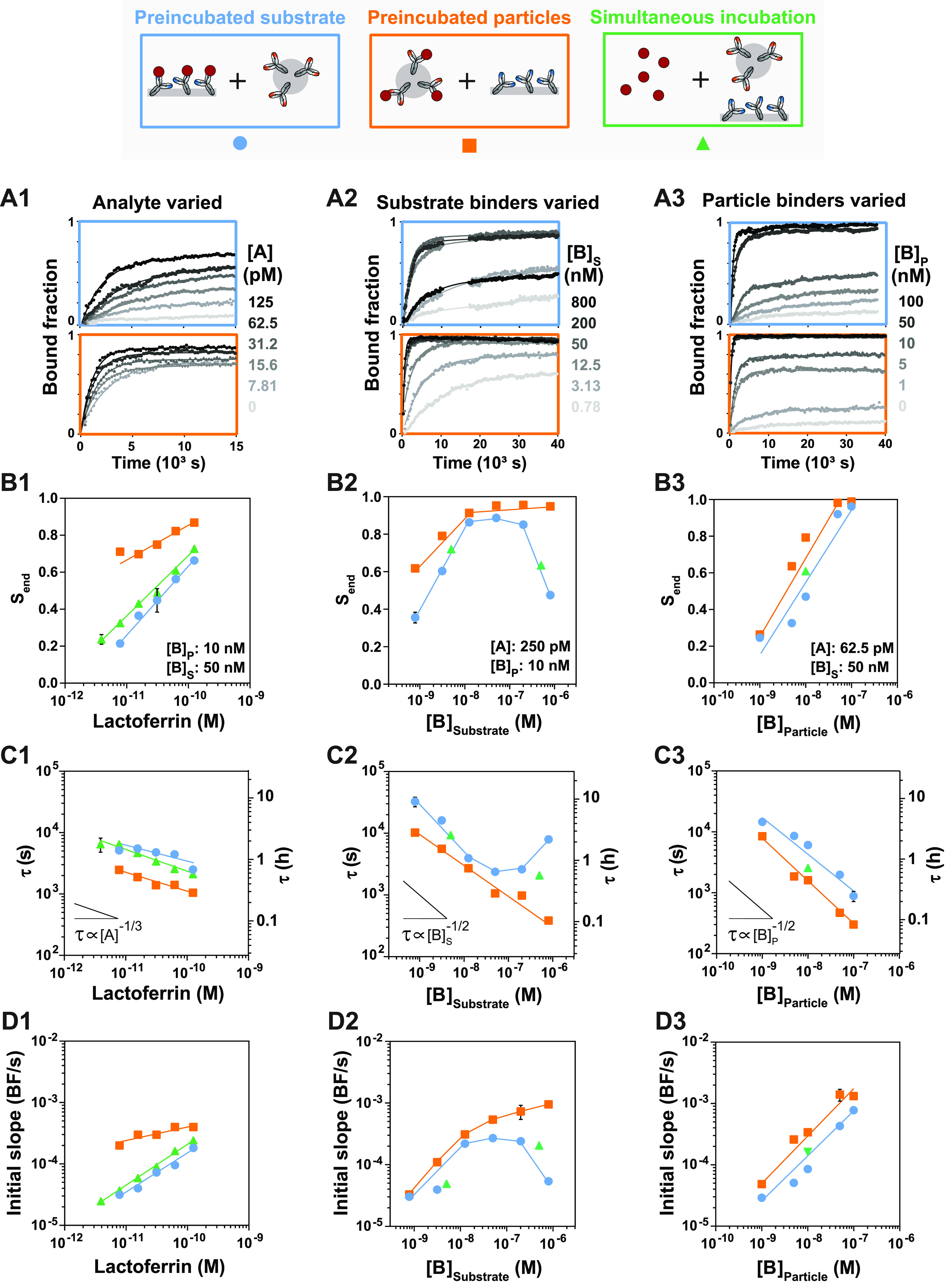
Distinguishing the reaction pathways of the sandwich immunosensor. Signal response over time for varying analyte concentrations (A1), substrate binder functionalization concentrations (A2), and particle binder functionalization concentrations (A3). Analyte was preincubated with the substrate (blue) and analyte was preincubated with the particles (orange). The maximal signal *S*_end_ (B1–3) and characteristic response time τ (C1–3) were extracted from the fits in (A1–3). The green data points were extracted from the simultaneous incubation measurements (Figure 3). (D1–3) The initial slope (Bound fraction/second) was extracted from linear fits of the first 2000 s (Supporting Information Figure S5). The colored lines are a guide to the eye and the error bars (not always visible) indicate the 95% confidence interval of the values extracted from the fits.

To study how pathways and the limiting reactions can be distinguished in the sensor response, an immunosensor design with fixed antibody densities (10 nM functionalized on particles, 50 nM functionalized on substrate) and a fixed chamber height (450 μm) was studied for three different incubation configurations, as indicated in Figure 5. The blue data refers to particles interacting with a preincubated substrate, in order to study reaction AS → SAP. The orange data refers to preincubated particles interacting with a substrate, in order to study reaction AP → SAP. The green data refers to particles and substrate simultaneously incubated with analyte, which is the mode of operation of the sandwich immunosensor (reactions are shown in Figure 1E, data are taken from Figure 3).

The sensor response was measured as a function of time for varying analyte concentrations (Figure 5A1), substrate binder functionalization concentrations (Figure 5A2), and particle binder functionalization concentrations (Figure 5A3), for the preincubated substrate configuration (blue) and the preincubated particles configuration (orange). Panels B–D show the three key parameters (maximal signal, characteristic response time, and initial slope) that were extracted from the time-dependent measurements shown in panel A and from Figure 3.

The preincubated particle experiments give the largest maximal signals (Figure 5B1), shortest characteristic response times (Figure 5C1), and largest initial slopes (Figure 5D1). The preincubated substrate experiments give the smallest maximal signals, longest characteristic response times, and smallest initial slopes. This indicates that when analyte binds to the particles first (pathway AP), a higher sensitivity and faster signal response are obtained compared to when analyte binds to the substrate first (pathway AS). This is because an analyte–particle complex can bind to any binder on the substrate, causing a high sandwich reaction rate (AP → SAP), while an analyte molecule on the substrate can bind only to a particle binder in close proximity, causing a lower sandwich reaction rate (AS → SAP).

The simultaneous incubation results are between the preincubated particles and preincubated substrate results and are always much closer to the preincubated substrate than to the preincubated particles results. To explain what this indicates, let us consider the reaction scheme in Figure 1E. In this reaction scheme, reactions occur in series and also in parallel. When reactions occur in series, the slowest reaction determines the reaction rate and reaction time. On the other hand, when reactions occur in parallel, the fastest reaction determines the reaction rate and time. Based on the data, we know that reaction AP → SAP is 2–3 times faster than reaction AS → SAP, which leaves the question, what are the time scales of reactions A → AP and A → AS? Since the number of binders and the density of the binders on the substrate are significantly higher than on the particles, we expect reaction A → AS to be much faster than A → AP. Taking this into consideration and knowing the characteristic response times of reactions AP → SAP and AS → SAP, we can match the trend observed in Figure 5C1 with the assumption that pathway AS is faster than pathway AP (see reaction pathway trends for varying characteristic response times of reactions A → AP and A → AS in Supporting Information Figure S8). Therefore, we conclude that AS is the dominant pathway in the immunosensor design with [B]_substrate_ = 50 nM and [B]_particle_ = 10 nM.

Increasing the binder density on the substrate (Figure 5A2) and on the particles (Figure 5A3) results in higher maximal signals (Figure 5B2,B3), lower characteristic response times (Figure 5C2,C3), and larger initial slopes (Figure 5D2,D3) for the preincubated substrate (AS → SAP) and preincubated particles (AP → SAP). Thus, a higher binder density on the substrate and/or the particles results in a more sensitive and faster immunosensor. However, an optimum substrate binder density is observed for the preincubated substrate. For [B]_substrate_ > 50 nM, a decrease in the maximal signal (Figure 5B2), an increase in τ (Figure 5C2), and a decrease in the initial slope (Figure 5D2) is observed. This trend was also observed before in the simultaneous incubation experiments shown in Figure 3. Interestingly, this optimum is not observed for particles preincubated with analyte, which confirms that higher effective binder densities are obtained when the substrate is prepared with [B]_substrate_ > 50 nM. The optimum substrate binder density is further studied and discussed in Supporting Information Figure S7.

As observed in Figures 2–5, the sensor response is influenced by all reactant concentrations (analyte, substrate binders, and particle binders). The preincubation experiments show that the characteristic response time of the sensor more strongly depends on the substrate binders (Figure 5C2) and particle binders (Figure 5C3) compared to the analyte (Figure 5C1). The fastest sensor response is obtained by increasing the binder density on the particles (Figure 5C3,D3).

## Conclusions and Outlook

We have examined how molecular concentrations and diffusion contribute to the time-dependent response of a sandwich immunosensor with particle labels and simultaneous antibody exposure with lactoferrin (80 kDa protein) as the model analyte. There are two pathways that lead to the formation of sandwich complexes: pathway AS and pathway AP. Pathway AS involves analyte binding to the substrate, followed by binding of a particle. Pathway AP involves binding of the analyte to a particle first and then to the substrate. Different sensor designs have been studied, showing characteristic response times that range from several hours to several minutes.

All measurement data of the sensor response over time can be fitted with single-exponential curves, where the system develops asymptotically from an initial state toward a final state. The extracted initial slope, characteristic time to reach the maximal signal value, and magnitude of the maximal signal were dependent on all three reactant concentrations: analyte, substrate binder, and particle binder. When the analyte binds first to the particles, a higher sensitivity and faster sensor response are obtained compared to the pathway where the analyte binds first to the substrate. For a concrete biosensor design, we found that the biosensor response is dominated by the reaction pathway in which analyte molecules bind first to the substrate and thereafter to a particle. Within this pathway, the binding of a particle to substrate-bound analyte dominates the sensor response time.

## Materials and Methods

### Materials

Transparent 96-well plates (Nunc MaxiSorb flat-bottom), Dynabeads MyOne Streptavidin C1, and bovine lactoferrin polyclonal antibody (A10–126A) were purchased from Thermo Fisher Scientific. PBS tablets, NaCl, bovine serum albumin (BSA), and bovine lactoferrin (L-047–50MG) were ordered from Sigma-Aldrich. Biotin-mPEG (MW 1 kDa) was obtained from Nanocs. Polystyrene slides (25 mm × 75 mm) were laser-cut from polystyrene sheets (transparent) obtained from Goodfellow. Custom-made fluid cell stickers with a surface area of 44 mm^2^ and height of 450 or 100 μm were purchased from Grace Biolabs.

### Functionalization of Particles

The streptavidin-coated Dynabeads (2 μL 10 mg/mL) were incubated with 2 μL of biotinylated polyclonal anti-lactoferrin antibody (concentration was varied) for 30 min at room temperature (RT) on a rotating fin (VWR, The Netherlands). Subsequently, 200 μL of 100 μM 1 kDa mPEG-biotin in PBS was added and incubated for 30 min at RT on the rotating fin. The particle mixture was put against a magnet to collect the particles and was washed two times with 500 μL of PBST (PBS with 0.05% Tween-20). The particles were resuspended in PBS with 1% BSA and incubated for 1 h at RT on the rotating fin. Finally, the particles were sonicated with 10 pulses at 70% amplitude with a 0.5 s duty cycle (Hielscher UIS250 V, Ultrasound Technology) and diluted 30 times in the assay buffer (PBS with 0.1% BSA and 350 mM NaCl).

### 96-Well Plate Preparation

The polyclonal anti-lactoferrin antibody was diluted to 50 nM in the carbonate coating buffer (0.05 M carbonate, pH 10) and 50 μL was added to each well of a 96-well plate. The plate was sealed and incubated for 1 h at RT. Next, the coating solution was removed and 100 μL of blocking buffer (PBS with 1% BSA) was added and incubated for 1 h at RT. Subsequently, the blocking solution was removed and 40 μL of the diluted particles was added. Lastly, 10 μL of lactoferrin (200 fM to 2 nM) was added, and the samples were incubated for 1 h before measuring.

### Flow Cell Preparation

The custom-made flow cell sticker was mounted on a polystyrene slide. One slide contained six flow cells. The height of the flow cell was 450 or 100 μm; the rest of the dimensions were the same. For the 450 μm flow cells, a volume of 50 μL was used for every addition, and for the 100 μm flow cells, a volume of 20 μL. All additions were done manually using a micropipette. The polyclonal anti-lactoferrin antibody was diluted in the carbonate coating buffer and added to each flow cell (concentration was varied). The residual liquid at the outlet was removed, and the flow cells were incubated for 1 h at RT in a humidity chamber. Subsequently, the blocking buffer was added to each flow cell and incubated for 1 h at RT in a humidity chamber. The blocking buffer was washed away by using the assay buffer. For the standard simultaneous incubation, the diluted particles were added first, and the flow cells were incubated for 30 min to sediment the particles. Next, the lactoferrin, diluted in the assay buffer, was added, and the flow cells were immediately measured repeatedly over time at RT. For the sequential assays, lactoferrin was first preincubated for 3 h at RT with either the substrate (flow cell) or the particles. Subsequently, particles were added to the preincubated flow cell or preincubated particles were added to a (not preincubated) flow cell, and the flow cells were immediately measured repeatedly over time at RT. A 6-tip multipipet was used for all fluid additions to the flow cells, to ensure equal timings of incubations in the six separate flow cells on a single slide. The six flow cells were measured in series, which caused for each flow cell a measurement periodicity of a few minutes. The inlets and outlets of the flow cells were sealed to prevent the evaporation of the fluid.

### Measurements

All measurements were performed using a custom-built bright-field microscope containing a motorized XY stage (ASR series; 100 mm × 120 mm travel (Zaber)). A 10× magnification (10× DIN achromatic finite intl standard objective (Edmund Optics)), simple 3 mm green led (12 V) and 3.2 MP camera (Flir BFS-U3–32S4M-C) with a field of view of 0.71 mm × 0.53 mm (effective pixel size 345 nm) were used to visualize the particles. A miniature linear actuator (Zaber T-LA13A) was used for the autofocus. The custom-built microscope was controlled using MATLAB. The positions to be measured were set (different flow cells on one slide or different wells of a 96-well plate), and each position was measured for 0.2 min at a framerate of 60 Hz. Multiple series of all positions were measured, allowing measurement of the response over time of six flow cells at once. The frames of each measurement were analyzed in real time using particle tracking software described by Bergkamp et al.^20^ The diffusivity time traces are obtained from the particle tracking data.^21−25^ The bound fraction is the output parameter that is derived from the diffusivity time traces (Supporting Information Figure S1).

## Supporting Information Available

Estimated sensor parameters, the BPM sensing principle, control experiments, linear fits of initial slopes, signal response in flow cells with a height of 100 μm, bound fraction at different positions within a single flow cell, and estimated reaction time scales (Figures S1–S8) (Table S1) (PDF)

## Abbreviations

BPM: biosensing by particle motion
BSA: bovine serum albumin

## References

[ref1] Zeng S.; Yong K. T.; Roy I.; Dinh X. Q.; Yu X.; Luan F. A Review on Functionalized Gold Nanoparticles for Biosensing Applications. Plasmonics 2011, 6 (3), 491–506. 10.1007/s11468-011-9228-1.

[ref2] Zhang Y.; Zhou D. Magnetic Particle-Based Ultrasensitive Biosensors for Diagnostics. Expert Rev. Mol. Diagn. 2012, 12 (6), 565–571. 10.1586/erm.12.54. 22845477

[ref3] Miyagawa A.; Okada T. Biosensing Strategies Based on Particle Behavior. Chemosensors 2023, 11 (3), 172 10.3390/chemosensors11030172.

[ref4] Chun P. Colloidal Gold and Other Labels for Lateral Flow Immunoassays. In Lateral Flow Immunoassay; Springer, 2009; pp 1–19.

[ref5] Ferrari E. Gold Nanoparticle-Based Plasmonic Biosensors. Biosensors 2023, 13 (3), 411 10.3390/bios13030411. 36979623 PMC10046074

[ref6] Bruls D. M.; Evers T. H.; Kahlman J. A. H.; Van Lankvelt P. J. W.; Ovsyanko M.; Pelssers E. G. M.; Schleipen J. J. H. B.; De Theije F. K.; Verschuren C. A.; Van Der Wijk T.; Van Zon J. B. A.; Dittmer W. U.; Immink A. H. J.; Nieuwenhuis J. H.; Prins M. W. J. Rapid Integrated Biosensor for Multiplexed Immunoassays Based on Actuated Magnetic Nanoparticles. Lab Chip 2009, 9 (24), 3504–3510. 10.1039/b913960e. 20024029

[ref7] Jamshaid T.; Neto E. T. T.; Eissa M. M.; Zine N.; Kunita M. H.; El-Salhi A. E.; Elaissari A. Magnetic Particles: From Preparation to Lab-on-a-Chip, Biosensors, Microsystems and Microfluidics Applications. TrAC, Trends Anal. Chem. 2016, 79, 344–362. 10.1016/j.trac.2015.10.022.

[ref8] Kim K.; Hall D. A.; Yao C.; Lee J. R.; Ooi C. C.; Bechstein D. J. B.; Guo Y.; Wang S. X. Magnetoresistive Biosensors with On-Chip Pulsed Excitation and Magnetic Correlated Double Sampling. Sci. Rep. 2018, 8 (1), 16493 10.1038/s41598-018-34720-0. 30405155 PMC6220270

[ref9] Zhao W.; Chiuman W.; Brook M. A.; Li Y. Simple and Rapid Colorimetric Biosensors Based on DNA Aptamer and Noncrosslinking Gold Nanoparticle Aggregation. ChemBioChem 2007, 8 (7), 727–731. 10.1002/cbic.200700014. 17410623

[ref10] Buskermolen A. D.; Lin Y.-T.; van Smeden L.; van Haaften R. B.; Yan J.; Sergelen K.; de Jong A. M.; Prins M. W. J. Continuous Biomarker Monitoring with Single Molecule Resolution by Measuring Free Particle Motion. Nat. Commun. 2022, 13 (1), 6052 10.1038/s41467-022-33487-3. 36229441 PMC9561105

[ref11] van Smeden L.; Saris A.; Sergelen K.; de Jong A. M.; Yan J.; Prins M. W. J. Reversible Immunosensor for the Continuous Monitoring of Cortisol in Blood Plasma Sampled with Microdialysis. ACS Sens. 2022, 7, 3041–3048. 10.1021/acssensors.2c01358. 36255855 PMC9623578

[ref12] Yan J.; van Smeden L.; Merkx M.; Zijlstra P.; W J Prins M. Continuous Small-Molecule Monitoring with a Digital Single-Particle Switch. ACS Sens. 2020, 5 (4), 1168–1176. 10.1021/acssensors.0c00220. 32189498 PMC8177406

[ref13] Zeng Q.; Zhou X.; Yang Y.; Sun Y.; Wang J.; Zhai C.; Li J.; Yu H. Dynamic Single-Molecule Sensing by Actively Tuning Binding Kinetics for Ultrasensitive Biomarker Detection. Proc. Natl. Acad. Sci. U.S.A. 2022, 119 (10), e2120379119 10.1073/pnas.2120379119. 35238650 PMC8916011

[ref14] Squires T. M.; Messinger R. J.; Manalis S. R. Making It Stick: Convection, Reaction and Diffusion in Surface-Based Biosensors. Nat. Biotechnol. 2008, 26 (4), 417–426. 10.1038/nbt1388. 18392027

[ref15] Lubken R. M.; Bergkamp M. H.; de Jong A. M.; Prins M. W. J. Sensing Methodology for the Rapid Monitoring of Biomolecules at Low Concentrations over Long Time Spans. ACS Sens. 2021, 6 (12), 4471–4481. 10.1021/acssensors.1c01991. 34854303 PMC8715529

[ref16] Rodbard D.; Feldman Y.; Jaffe M. L.; Miles L. E. M. Kinetics of Two-Site Immunoradiometric (’sandwich’) Assays-II. Studies on the Nature of the -High-Dose Hook Effect. Immunochemistry 1978, 15 (2), 77–82. 10.1016/0161-5890(78)90046-9. 631868

[ref17] Lin C. H.; Chen H. Y.; Yu C. J.; Lu P. L.; Hsieh C. H.; Hsieh B. Y.; Chang Y. F.; Chou C. Quantitative Measurement of Binding Kinetics in Sandwich Assay Using a Fluorescence Detection Fiber-Optic Biosensor. Anal. Biochem. 2009, 385 (2), 224–228. 10.1016/j.ab.2008.11.010. 19041630

[ref18] Rey E. G.; O’Dell D.; Mehta S.; Erickson D. Mitigating the Hook Effect in Lateral Flow Sandwich Immunoassays Using Real-Time Reaction Kinetics. Anal. Chem. 2017, 89 (9), 5095–5100. 10.1021/acs.analchem.7b00638. 28388030 PMC5839149

[ref19] Karunakaran C.; Pandiaraj M.; Santharaman P. Immunosensors. In Biosensors and Bioelectronics; Elsevier Inc, 2015.

[ref20] Bergkamp M. H.; Cajigas S.; van IJzendoorn L. J.; Prins M. W. J. High-Throughput Single-Molecule Sensors: How Can the Signals Be Analyzed in Real Time for Achieving Real-Time Continuous Biosensing. ACS Sens. 2023, 8, 2271–2281. 10.1021/acssensors.3c00245. 37216442 PMC10294250

[ref21] Vashist S. K.; Dixit C. K.; MacCraith B. D.; O’Kennedy R. Effect of Antibody Immobilization Strategies on the Analytical Performance of a Surface Plasmon Resonance-Based Immunoassay. Analyst 2011, 136 (21), 4431–4436. 10.1039/c1an15325k. 21904732

[ref22] Trilling A. K.; Beekwilder J.; Zuilhof H. Antibody Orientation on Biosensor Surfaces: A Minireview. Analyst 138, 1619 1627. 10.1039/c2an36787d. 23337971

[ref23] Kausaite-Minkstimiene A.; Ramanaviciene A.; Kirlyte J.; Ramanavicius A. Comparative Study of Random and Oriented Antibody Immobilization Techniques on the Binding Capacity of Immunosensor. Anal. Chem. 82, 6401 6408. 10.1021/ac100468k. 20669994

[ref24] Gao S.; Guisán J. M.; Rocha-Martin J. Oriented Immobilization of Antibodies onto Sensing Platforms - A Critical Review. Anal. Chim. Acta 2022, 1189, 338907 10.1016/j.aca.2021.338907. 34815045

[ref25] Lin Y.-T.; Vermaas R.; Yan J.; de Jong A. M.; Prins M. W. J. Click-Coupling to Electrostatically Grafted Polymers Greatly Improves the Stability of a Continuous Monitoring Sensor with Single-Molecule Resolution. ACS Sens. 2021, 6 (5), 1980–1986. 10.1021/acssensors.1c00564. 33985333 PMC8165697

