# Supplementary material for: Sandwich Immunosensor Based on Particle Motion: How Do Reactant Concentrations and Reaction Pathways Determine the Time-Dependent Response of the Sensor?
Source: ACS Sens. 2023 Nov 13;8(11):4216–25. doi: 10.1021/acssensors.3c01549 (PMC10683507; doi:10.1021/acssensors.3c01549)
Supplement: Supplementary file 1 — se3c01549_si_001.pdf [file se3c01549_si_001.pdf]

# Supporting information

## Sandwich immunosensor based on particle motion: how do reactant concentrations and reaction pathways determine the time-dependent response of the sensor?

Claire M. S. Michielsen[a,c], Alissa D. Buskermolen[a,c], Arthur M. de Jong[b,c], Menno W.J. Prins[a,b,c,d]*

[a] *Department of Biomedical Engineering, Eindhoven University of Technology, Eindhoven 5612 AE, The Netherlands*

[b] *Department of Applied Physics, Eindhoven University of Technology, Eindhoven 5612 AE, The Netherlands*

[c] *Institute for Complex Molecular Systems (ICMS), Eindhoven University of Technology, Eindhoven 5612 AE, The Netherlands*

[d] *Helia Biomonitoring, Eindhoven 5612 AR, The Netherlands*

*Email: m.w.j.prins@tue.nl

## Contents

1. List of Supporting Information Figures and Tables ........ 2

2. Estimated parameters of the sandwich immunosensor ........ 3

3. Biosensing by Particle Motion – sensing principle and readout parameters ........ 4

4. Positive and negative controls of the kinetic measurements in flow cells ........ 5

5. Influence of washing on the signal response ........ 5

6. Linear fits of initial slopes ........ 6

7. Signal response in flow cells with a height of 100 µm ........ 7

8. Bound fraction at different positions within a flow cell for varying substrate binder concentrations ........ 7

9. Estimation of reaction timescales ........ 8

# 1. List of Supporting Information Figures and Tables

## Supporting Information Figures

**Figure S1.** Biosensing by Particle Motion, sensing principle and readout parameters. ……………..…4

**Figure S2.** Signal response for varying substrate binder densities using particles with and without binder molecules (antibodies). ……………………………………………………………………………5

**Figure S3** Signal response in the presence of Lactoferrin with several washing steps………............5

**Figure S4.** Initial slopes of signal response for varying analyte and substrate binder concentrations.………………………………………………………………………………………………6

**Figure S5.** Initial slopes of experiments with preincubation.……………………………………..……… 6

**Figure S6.** Signal response for varying analyte and substrate binder concentrations obtained in flow cells with a height of 100 µm…………..……………………………………………………………7

**Figure S7.** Bound fraction measured at five different positions within six different flow cells, where each flow cell was prepared with a different substrate binder concentration…..……………………….…7

**Figure S8.** Timescales of the reactions leading to the formation of sandwich complexes…………..…8

## Supporting Information Tables

**Table S1.** Estimated sensor parameters………………..……………………………………............... 3

## 2. Estimated parameters of the sandwich immunosensor

***Table S1. Estimated sensor parameters***

| Parameter | Value/estimation |
|---|---|
| Flow cell 1 | |
| - Height (H) | 450 μm |
| - Volume | 20 μL |
| Flow cell 2 | |
| - Height (H) | 100 μm |
| - Volume | 4.4 μL |
| **Surface area** | |
| Fluid cell, bottom surface with particles | $44\ \mathrm{mm}^2$ |
| Fluid cell, top surface plus bottom surface | $88\ \mathrm{mm}^2$ |
| Microscope field of view | $0.374\ \mathrm{mm}^2$ |
| 1 μm particle, single particle surface area | $3.14 \cdot 10^{-6}\ \mathrm{mm}^2$ |
| Antibody, projected surface area | $56 - 119 \cdot 10^{-12}\ \mathrm{mm}^2$ (tail-on vs flat-on) |
| **DynaBeads MyOne C1 Streptavidin particles** | |
| Diameter | 1.0 μm |
| Particle concentration (stock) | $10\ \mathrm{mg\ mL}^{-1}$<br>$9 \cdot 10^9$ particles $\mathrm{mL}^{-1}$ |
| Particle concentration in flow cell<br>- Flow cell 1: stock 3000x diluted<br>- Flow cell 2: stock 600x diluted | $3.3\ \mathrm{mg\ mL}^{-1}$<br>$3 \cdot 10^6$ particles $\mathrm{mL}^{-1}$<br>$1.5 \cdot 10^7$ particles $\mathrm{mL}^{-1}$ |
| Binding capacity (specified by supplier) | 20 μg biotinylated Ab per mg particle<br>(0.4 μg biotinylated Ab per 2 μL particles) |
| **Substrate-side binders** | |
| Number of Ab that fit theoretically in the flow cell (top and bottom; based on surface areas) | $7 - 16 \cdot 10^{11}$ molecules per flow cell (tail-on vs flat-on) |
| Number of Ab incubated using 50 nM | $6.0 \cdot 10^{11}$ molecules ($1 \cdot 10^{-14}$ mol) |
| **Particle-side binders** | |
| Number of particles per flow cell (1&2) | 60 000 particles |
| Average number of particles in FOV | 500 particles |
| Number of Ab that fit theoretically on a particle (based on surface areas) | $2.5 - 5.4 \cdot 10^4$ molecules per particle (tail-on vs flat-on) |
| Maximum number of Ab on a particle (2 μL particles from stock + 2 μL 10 nM biotin-antibody) | ~600 molecules per particle |
| Maximum number of Ab on particles in flow cell | $4 \cdot 10^7$ molecules on particles in flow cell |
| Total surface area of all particles in a flow cell (20 μL) | $\sim 0.2\ \mathrm{mm}^2$ |
| **Analyte** | |
| Molecular weight of lactoferrin | 80 kDa |
| Stokes radius[1] | |
| - Monomer | 2.2 nm |
| - Tetramer | 4.4 nm |
| Diffusion coefficient[1] | |
| - Monomer | $9.6 \cdot 10^{-11}\ \mathrm{m}^2\ \mathrm{s}^{-1}$ |
| - Tetramer | $4.9 \cdot 10^{-11}\ \mathrm{m}^2\ \mathrm{s}^{-1}$ |
| Diffusion time: $\tau_D = H^2/D$ (H = 450μm) | |
| - Monomer | $2109\ \mathrm{s} = 35\ \mathrm{min}$ |
| - Tetramer | $4133\ \mathrm{s} = 69\ \mathrm{min}$ |
| Number of molecules in flow cell 1 (20 μL) | |
| - 250 pM | $3.0 \cdot 10^9$ molecules |
| - 62.5 pM | $7.5 \cdot 10^8$ molecules |
| - 7.81 pM | $9.4 \cdot 10^7$ molecules |

[1.] Bernard Chaufer,*et al*. Selective extraction of lysozyme from a mixture with lactoferrin by ultrafiltration. Role of the physico-chemical environment. Le Lait, 2000, 80 (1), pp.197-203. 10.1051/lait:2000119. hal-00895400

## 3. Biosensing by Particle Motion – sensing principle and readout parameters

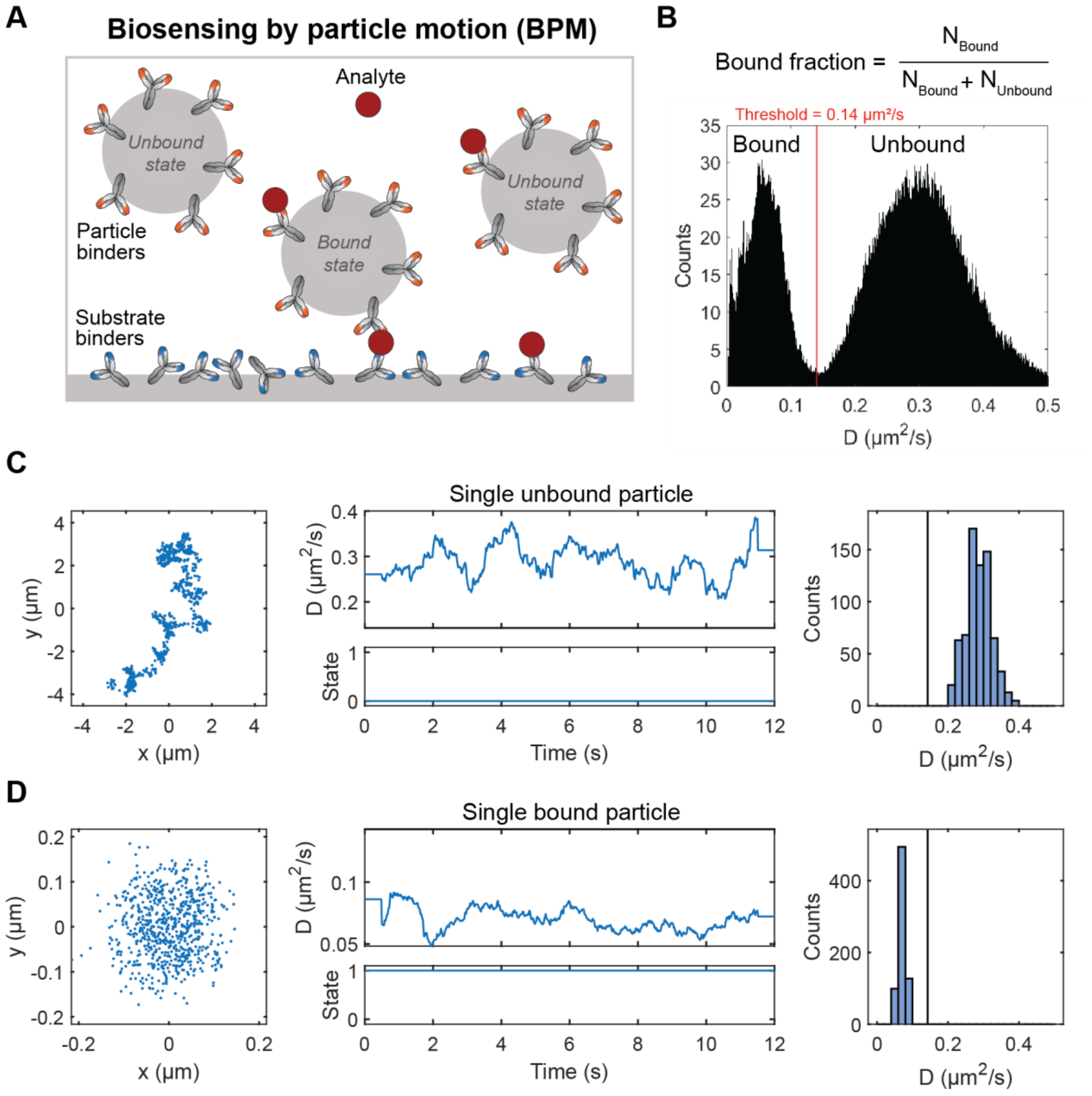


**Figure S1. Biosensing by Particle Motion, sensing principle and readout parameters.** (A) Sketch of the sandwich immunosensor with antibodies immobilized on the substrate and particles. The particles hover over the substrate and experience free Brownian motion, which is referred to as the unbound state. In the presence of the analyte molecule, a particle can transition into a bound state, which results in a confined motion. (B) Diffusivity distribution of all particles (~600). A threshold at D = 0.14 µm$^2$/s is used to differentiate between bound and unbound states. The bound fraction is defined as the ratio between the population of bound states and the total population of states during the measurement time. (C) 2-D motion pattern (left), diffusivity time trace (middle) and diffusivity distribution (right) of a single particle in the unbound state. (D) 2-D motion pattern (left), diffusivity time trace (middle) and diffusivity distribution (right) of a single particle in the bound state.

## 4. Positive and negative controls of the kinetic measurements in flow cells

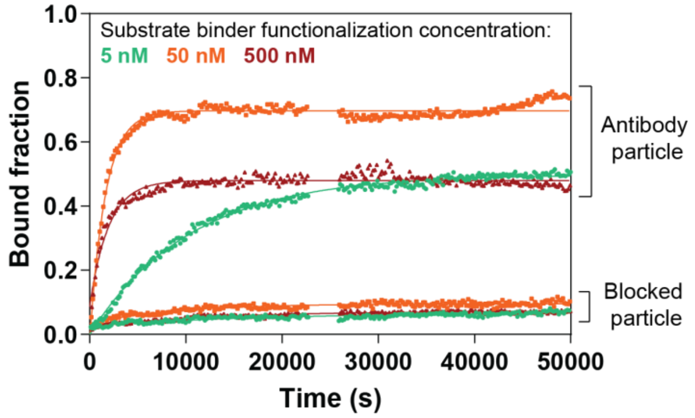


**Figure S2. Signal response for varying substrate binder densities using particles with and without binder molecules (antibodies).** The polystyrene substrates were functionalized by physisorption of antibodies using concentrations of 5 nM (green), 50 nM (orange) and 500 nM (red). Particles were prepared using 10 nM Ab or no antibodies; in both cases, the particles were blocked using biotin-PEG and BSA. The negative controls (blocked particles) show no response in the presence of 250 pM Lactoferrin and the positive controls (particles with antibodies) show a significant signal increase over time. The data were fitted with single-exponential curves according to Equation 1.

## 5. Influence of washing on the signal response

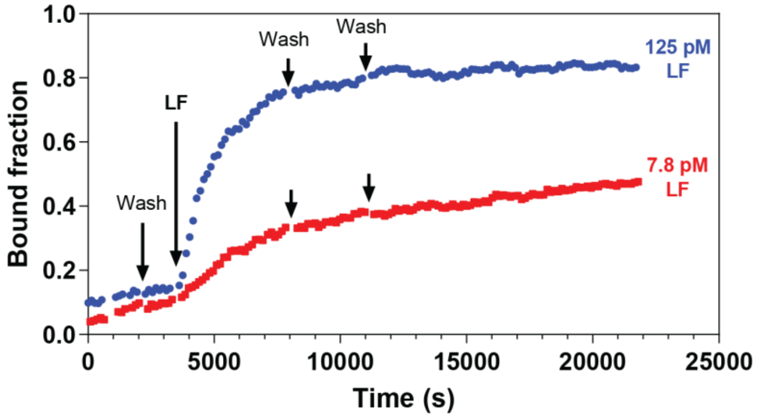


**Figure S3. Signal response in the presence of Lactoferrin with several washing steps.** The substrates were functionalized with 50 nM antibodies and the particles with 10 nM antibodies. 125 pM (blue) and 7.8 pM (red) lactoferrin was added to the flow cell after 3600 seconds (indicated with black arrow LF). Several washing steps (50 µL assay buffer; indicated with black arrow Wash) were applied. After the washing steps, no decreases in bound fraction were observed.

## 6. Linear fits of initial slopes

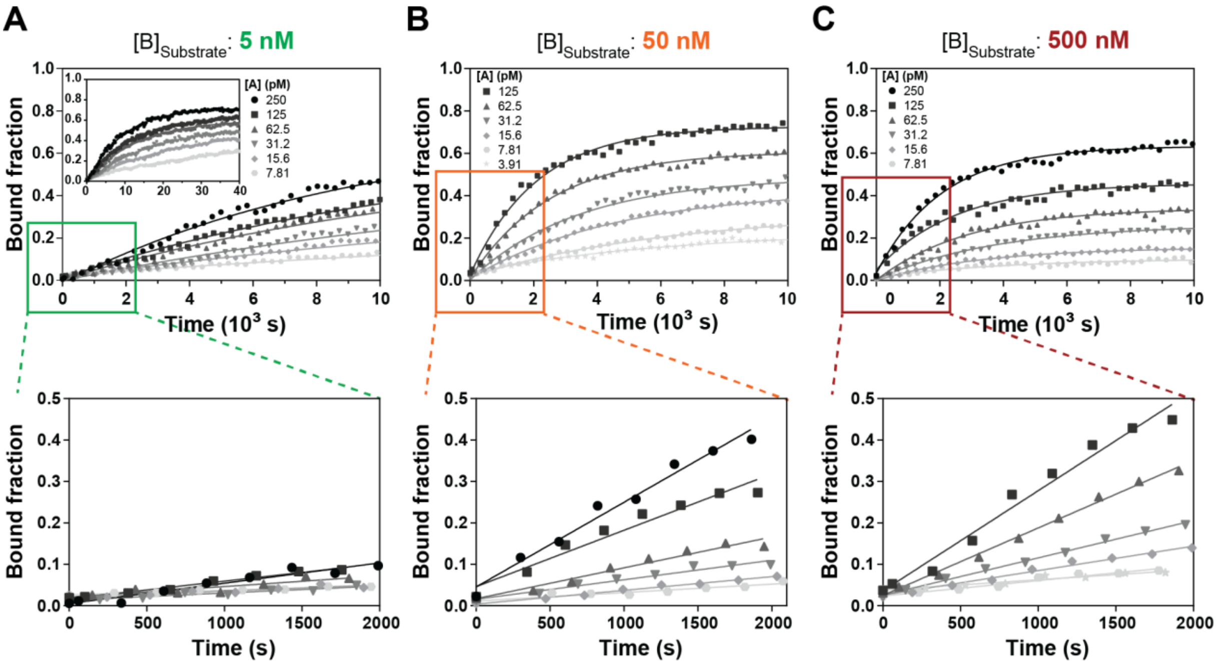


**Figure S4. Initial slopes of signal response for varying analyte and substrate binder concentrations.** The signal of the BPM sensor as a function of time, studied in a static flow cell for different lactoferrin concentrations. The polystyrene substrates were functionalized by physisorption of antibodies using concentrations of 5 nM (A), 50 nM (B) and 500 nM (C). At the top, the data were fitted with single-exponential curves according to Equation 1. At the bottom, the data of the first 2000 seconds were fitted with a linear equation to obtain the initial rates.

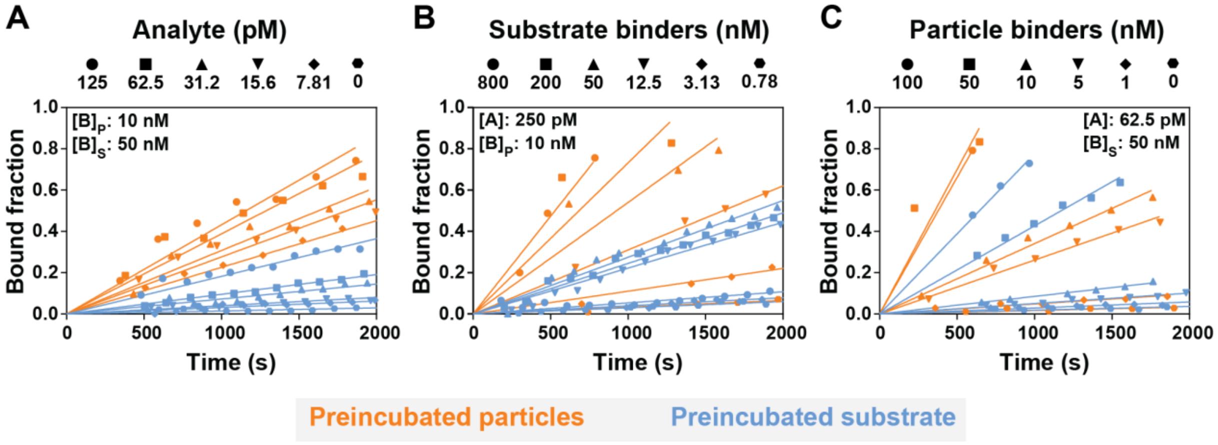


**Figure S5. Initial slopes of experiments with preincubation.** Signal response of the first 500-2000 seconds for (A) varying analyte concentrations, (B) varying substrate binder functionalization concentrations, and (C) varying particle binder functionalization concentrations. Analyte was preincubated with the substrate (blue) and analyte was preincubated with the particles (orange). The data were fitted with a linear equation to obtain the initial rates (solid lines).

## 7. Signal response in flow cells with a height of 100 µm

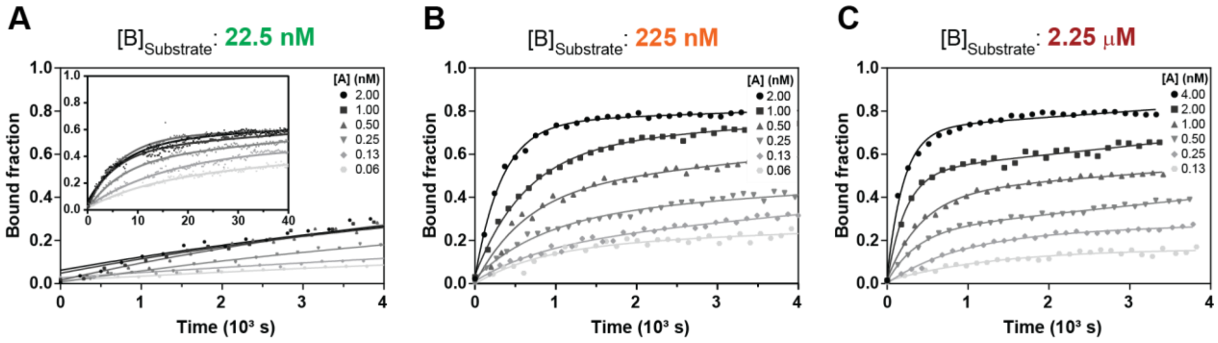


**Figure S6. Signal response for varying analyte and substrate binder concentrations obtained in flow cells with a height of 100 µm.** The polystyrene substrates were functionalized by physisorption of antibodies using concentrations of (A) 22.5 nM, (B) 225 nM, and (C) 2.25 µM. The data were fitted with single-exponential curves according to Equation 1 (solid lines).

## 8. Bound fraction at different positions within a flow cell for varying substrate binder concentrations

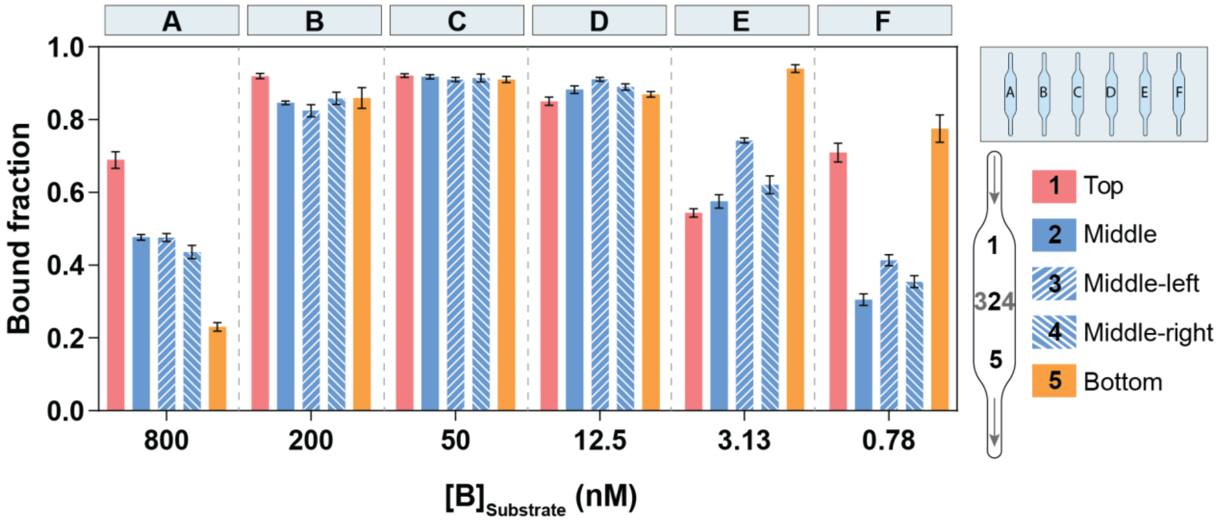


**Figure S7. Bound fraction measured at five different positions (1-5, indicated as bars with different colors) within six different flow cells on a single slide (flow cells A-F), where each flow cell was prepared with a different substrate binder concentration (from A to F: 800, 200, 50, 12.5, 3.13, 0.78 nM, respectively).** The bound fraction was measured after 17 hours at 5 different positions in each flow cell: (1) near the inlet, (2) in the middle, (3) left from the middle, (4) right from the middle, and (5) near the outlet. 250 pM lactoferrin was preincubated on the substrate and the particles were prepared using 10 nM antibodies. The error bars depict the standard deviation of the mean of 10 measurements at a given position within a given flow cell, recorded over a period of three hours.

Comparing different positions within the flow cell of the preincubated substrate reveals that a higher bound fraction is obtained near the inlet of the flow cell compared to the middle and the outlet of the flow cell for high $[B]_{substrate}$ (Supporting Information Fig. S7). This indicates that the analyte molecules are captured more efficiently at the inlet of the flow cell for high substrate binder densities, which results in less analyte molecules in the field of view and therefore a lower bound fraction and slower signal response. For $[B]_{substrate}$ = 50 nM, a uniform bound fraction is observed for all five positions. Decreasing the binder density further results in a less homogenous bound fraction, which could be due to larger variations in the physisorption of binder molecules at low concentrations.

## 9. Estimation of reaction timescales

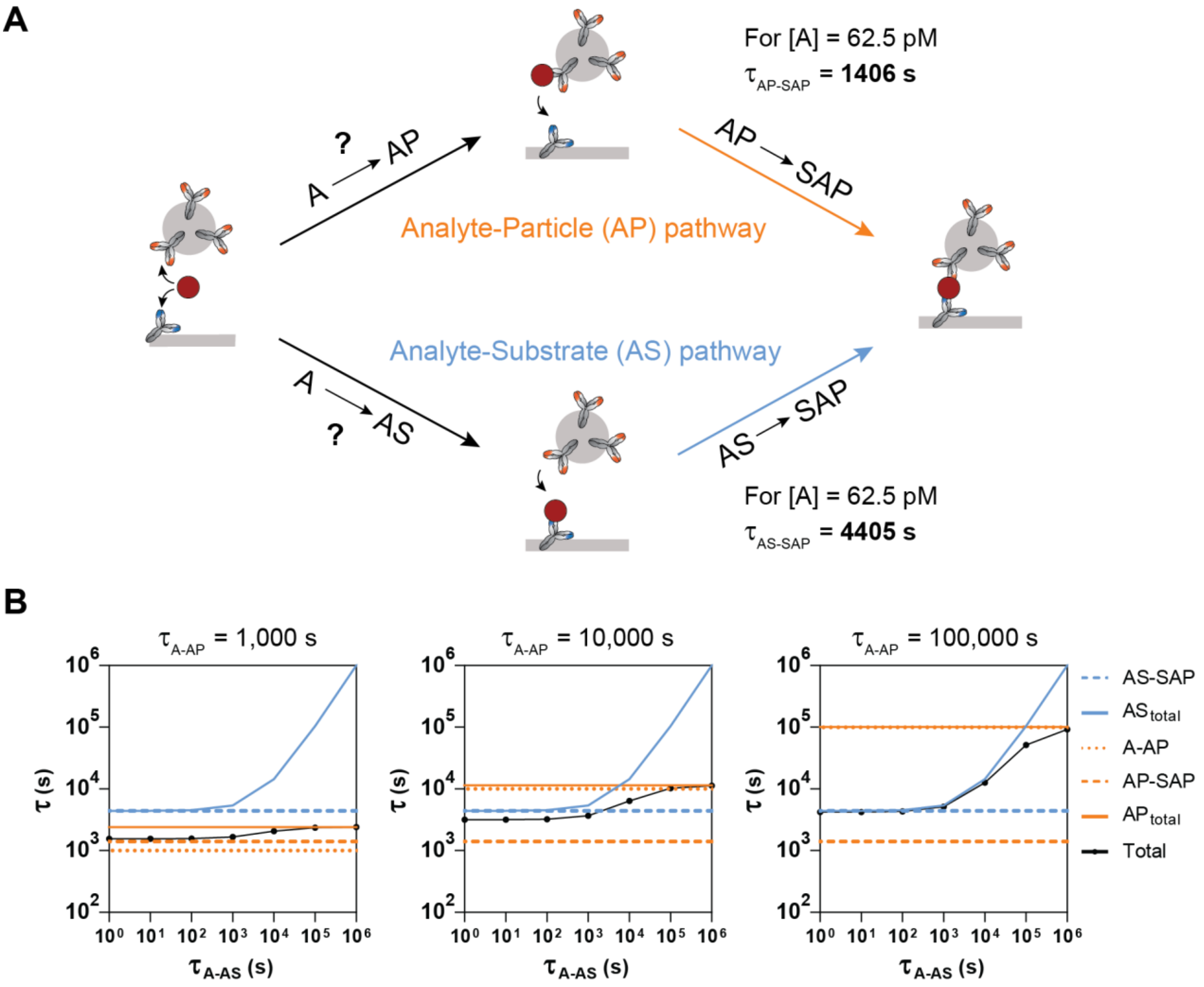


**Figure S8. Timescales of the reactions leading to the formation of sandwich complexes.** (A) Reaction scheme of an irreversible sandwich immunosensor. The analyte can first bind to the particle (A→AP) or to the substrate (A→AS), subsequently the analyte-particle complex can bind to the substate (AP→SAP) or a particle binds to the analyte-substrate complex (AS→SAP). The reactions A→AP and AP→SAP occur in series (pathway AP), thus the total characteristic response time of pathway AP is the sum of both reactions: $\tau_{AP_{total}} = \tau_{A-AP} + \tau_{AP-SAP}$. The reactions A→AS and AS→SAP (pathway AS) also occur in series, so $\tau_{AS_{total}} = \tau_{A-AS} + \tau_{AS-SAP}$. Pathway AP and AS occur in parallel, therefore the characteristic response time $\tau_{total}$ of the total scheme of Fig. S8A is given by: $\frac{1}{\tau_{total}} = \frac{1}{\tau_{AP_{total}}} + \frac{1}{\tau_{AS_{total}}} \rightarrow \tau_{total} = \frac{1}{\frac{1}{\tau_{A-AP}+\tau_{AP-SAP}} + \frac{1}{\tau_{A-AS}+\tau_{AS-SAP}}}$. The values of $\tau_{AP-SAP}$ and $\tau_{AS-SAP}$ are known from the preincubated experiments for varying analyte concentrations. $\tau_{A-AP}$ and $\tau_{A-AS}$ are unknown. (B) The characteristic response time of $\tau_{AP_{total}}$ of pathway AP (orange solid line), $\tau_{AS_{total}}$ of pathway AS (blue solid line) and $\tau_{total}$ of the total reaction (black solid line) are calculated based on the known values for $\tau_{AP-SAP}$ (orange dashed line) and $\tau_{AS-SAP}$ (blue dashed line). In these panels, $\tau_{A-AS}$ and $\tau_{A-AP}$ are varied, with $\tau_{A-AS}$ on the x-axis, for $\tau_{A-AP}$ = 1,000 s (left panel), 10,000 s (middle panel), and 100,000 s (right panel).

Fig. S8 sketches a reaction timescale model to estimate how the total reaction time of sandwich formation is influenced by the different sub-reactions. See the figure caption for explanations.

In Fig. 5C1, the results of the simultaneous incubation (total reaction) are in between the preincubated particles and preincubated substrate results, and always much closer to the preincubated substrate than to the preincubated particles results. Comparing these experimental results with the estimations in Fig. S8, we hypothesize that $\tau_{A-AP} > \tau_{A-AS}$ , $\tau_{A-AS} < 1{,}000\ s$ and $\tau_{A-AP}$ is of the order of 10,000 s.
